# Identifying dynamic, partially occupied residues using anomalous scattering

**DOI:** 10.1107/S2059798319014475

**Published:** 2019-11-19

**Authors:** Serena Rocchio, Ramona Duman, Kamel El Omari, Vitaliy Mykhaylyk, Christian Orr, Zhen Yan, Loïc Salmon, Armin Wagner, James C. A. Bardwell, Scott Horowitz

**Affiliations:** a Department of Molecular, Cellular and Developmental Biology, and the Howard Hughes Medical Institute, University of Michigan, Ann Arbor, MI 48109, USA; b Department of Biochemical Sciences ‘A. Rossi Fanelli’, Sapienza University of Roma, 00185 Rome, Italy; c Diamond Light Source Ltd, Diamond House, Harwell Science and Innovation Campus, Didcot OX11 0DE, England; d Research Complex at Harwell, Rutherford Appleton Laboratory, Harwell Oxford, Didcot OX11 0FA, England; e Centre de RMN à Très Hauts Champs, CNRS, ENS Lyon, UCB Lyon 1, Université de Lyon, 69100 Villeurbanne, France; f Department of Chemistry and Biochemistry and the Knoebel Institute for Healthy Aging, University of Denver, Denver, CO 80208, USA

**Keywords:** anomalous scattering, protein dynamics, partially occupied residues, crystallography, chaperones, protein folding

## Abstract

Although often presented as taking single ‘snapshots’ of the conformation of a protein, X-ray crystallography provides an averaged structure over time and space within the crystal. The important but difficult task of characterizing structural ensembles in crystals is typically limited to small conformational changes, such as multiple side-chain conformations. A crystallographic method was recently introduced that utilizes residual electron and anomalous density (READ) to characterize structural ensembles encompassing large-scale structural changes. Key to this method is an ability to accurately measure anomalous signals and distinguish them from noise or other anomalous scatterers. This report presents an optimized data-collection and analysis strategy for partially occupied iodine anomalous signals. Using the long-wavelength-optimized beamline I23 at Diamond Light Source, the ability to accurately distinguish the positions of anomalous scatterers with occupancies as low as ∼12% is demonstrated. The number and positions of these anomalous scatterers are consistent with previous biophysical, kinetic and structural data that suggest that the protein Im7 binds to the chaperone Spy in multiple partially occupied conformations. Finally, READ selections demonstrate that re-measured data using the new protocols are consistent with the previously characterized structural ensemble of the chaperone Spy with its client Im7. This study shows that a long-wavelength beamline results in easily validated anomalous signals that are strong enough to be used to detect and characterize highly disordered sections of crystal structures.

## Abstract 2

Structural studies of partially occupied, heterogeneous protein systems using crystallography are difficult. Here, methods are presented for detecting these states in crystals.

## 1. Introduction   

In crystallography, anomalous scattering is commonly used to help solve the phase problem (Hendrickson, 2014 ▸). A second, less well utilized aspect of anomalous scattering is its ability to selectively label and identify residues of interest. Crystallo­graphers can use anomalous maps to pinpoint metal ions (Handing *et al.*, 2018 ▸) or to aid in model building or electron-density interpretation (Pflug *et al.*, 2012 ▸; Wang *et al.*, 2012 ▸). We recently introduced a method called residual electron and anomalous density (READ) that uses anomalous maps to allow the reconstruction of highly heterogeneous conformational ensembles in regions of the protein that are not sufficiently well ordered for traditional model building (Salmon *et al.*, 2018 ▸; Horowitz *et al.*, 2016 ▸). This method uses selective anomalous labeling with iodophenylalanine (pI-Phe) to generate multiple partially occupied iodine anomalous signals in the crystal corresponding to different protein conformations. Ensemble-selection techniques (Venditti *et al.*, 2016 ▸; Salmon *et al.*, 2018 ▸; Horowitz *et al.*, 2016 ▸) are then used to create ensembles that are consistent with both the anomalous data and weak electron-density data for the disordered segment(s) of the crystal. The Im7 ensembles generated by READ were highly consistent with orthogonal experimental approaches that were also used to characterize Im7 binding and folding with Spy, including NMR spectroscopy and thermodynamic and kinetic analyses (Salmon *et al.*, 2018 ▸; Horowitz *et al.*, 2016 ▸; Koldewey *et al.*, 2016 ▸; Stull *et al.*, 2016 ▸; He & Hiller, 2018 ▸; He *et al.*, 2016 ▸).

A critical step in the READ process is the correct identification of weak anomalous signals (Horowitz, Salmon *et al.*, 2018 ▸; Wang, 2018 ▸). Selective labeling requires tunable X-ray sources to achieve high levels of anomalous scattering for particular atoms (Phillips *et al.*, 1976 ▸). However, many important anomalous scatterers do not have their *K* and *L* absorption edges within the wavelength ranges of standard synchrotron beamlines for macromolecular crystallography, resulting in weak anomalous signals. Recently, a novel beamline, I23, at Diamond Light Source was introduced to collect anomalous data at substantially longer wavelengths than was previously possible. The I23 beamline operates in an in-vacuum sample environment and features a number of other innovations that make it optimal for anomalous data collection (Wagner *et al.*, 2016 ▸). Here, we present an optimized data-collection strategy for the identification of iodine anomalous scattering positions that greatly improves our ability to detect weak and partially occupied states in crystals. Co-crystals of the chaperone Spy with its disordered client Im7 were used to collect anomalous data for three iodine-containing Im7 mutants. Using multiple wavelengths and data collections at different orientations obtained using a multi-axis goniometer, we demonstrate that it is possible to detect the iodine signal even at low occupancy and clearly distinguish it from other anomalous scatterers. The anomalous signals indicate that Im7 binds to Spy in multiple conformations along its concave surface, consistent with the findings from other biophysical investigations (Salmon *et al.*, 2018 ▸; Horowitz *et al.*, 2016 ▸). These data were used in a new READ selection, which yielded results consistent with our previously determined structural ensemble (Horowitz *et al.*, 2016 ▸). The data demonstrate the feasibility of using anomalous scatterers as an approach to understand disordered systems in crystallography.

## 2. Methods   

### 2.1. Crystallization   

The Super Spy chaperone mutant Spy H96L (Quan *et al.*, 2014 ▸) was used here in co-crystallization experiments, as we found that it formed crystals more readily than the wild-type Spy protein (Horowitz *et al.*, 2016 ▸). Spy–Im7 co-crystals were obtained using the vapor-diffusion technique with crystallization conditions consisting of 22–34% PEG 3000, 70–270 m*M* zinc acetate, 0.1 *M* imidazole pH 8.0. Crystallization was performed using 15–50 mg ml^−1^ Spy H96L with Im7 peptides that represent the 6–26 portion of the Im7 peptide in a 1:1–1:2 ratio. The Im7 (^6^SISDYTEAEFVQLLKEIEKEN^26^) peptides used here in the co-crystallization experiments have a single iodophenylalanine substitution at position 18 (L18pI-Phe), 19 (L19pI-Phe) or 20 (K20pI-Phe). Of note, we previously found that under these conditions Spy and its mutants only form diffraction-quality crystals in the presence of clients (casein and Im7 were previously tested), precluding the use of soaks (Horowitz *et al.*, 2016 ▸).

#### 2.1.1. Crystal harvesting   

The crystals were cryoprotected by increasing the PEG 3000 concentration to 35% and were flash-cooled in liquid nitrogen. The crystals were harvested using LithoLoop sample mounts (Molecular Dimensions, Newmarket, England) matched to the size of the crystal and glued to copper pins as part of the dedicated I23 sample-holder assembly, which is optimized for cryogenic sample transfer and storage in the beamline vacuum environment.

#### 2.1.2. Data collection   

Data were collected on beamline I23 at Diamond Light Source at X-ray energies of 5.2 and 4.5 keV, which are above and below the iodine *L* absorption edges [*E*(*L*_I_) = 5.188 keV, *E*(*L*_II_) = 4.852 keV, *E*(*L*_III_) = 4.557 keV], using the semi-cylindrical PILATUS 12M area detector. For each data set, 360° of data were collected with an exposure time of 0.1 s per 0.1° rotation in an inverse-beam setting of 20° wedges. Taking advantage of the multi-axis goniometer, data were collected at 5.2 keV at different κ and φ goniometer angles (Table 1 ▸). To identify the remaining anomalously scattering ligands, zinc and chloride ions, two additional sets of data were collected for the Spy–Im7 L19pI-Phe complex at 2.87 and 2.75 keV.

#### 2.1.3. Model building and refinement   

Data integration and scaling were performed with *XDS* (Kabsch, 2010 ▸) and with *AIMLESS* (Evans & Murshudov, 2013 ▸), respectively. Phased anomalous difference Fourier maps were calculated using *ANODE* (Thorn & Sheldrick, 2011 ▸). Molecular replacement was performed using the known structure of the Spy–Im7 complex (PDB entry 5wnw; Horowitz *et al.*, 2016 ▸) as the search model in *Phenix* (Adams *et al.*, 2010 ▸). Model building and refinement were accomplished using *Coot* (Emsley *et al.*, 2010 ▸) and *Phenix* (Afonine *et al.*, 2012 ▸), respectively. I atoms were placed into the peaks of the phased anomalous difference Fourier maps produced by *ANODE*. To test the stability and reproducibility of the iodine occupancy and atomic displacement parameter (ADP) refinements, we followed the protocol described in Langan *et al.* (2018 ▸). A total of 10 000 models with randomly assigned starting values were created using *phenix.pdb_tools*. 50 cycles of refinement using *phenix.refine* were performed with fixed *xyz* coordinates, refining only the occupancies and ADPs. The values in the final coordinate files were then based on the analysis of 2D histograms for the individual atoms. The protein structures were very similar in each case, with a maximum C^α^ root-mean-square deviation (r.m.s.d.) of 0.41 Å. Refinement statistics are given in Table 2 ▸.

#### 2.1.4. Dose estimation   

The absorbed dose was estimated for each crystal using *RADDOSE*-3*D* (Bury *et al.*, 2018 ▸). *RADDOSE*-3*D* calculates the dose absorbed by a crystal in an X-ray beam as function of the beam characteristics (wavelength, flux, profile and size), crystal properties (unit cell, size, amino-acid and solvent composition) and data-collection strategy (number of images, oscillation range and exposure time). The cumulative dose was estimated for those crystals submitted to multiple and sequential data collections.

### 2.2. READ ensemble selection   

The previously described READ selection procedure was used to select an ensemble of structures to fit the weak residual density and the iodine anomalous signals (Salmon *et al.*, 2018 ▸; Horowitz *et al.*, 2016 ▸). In order to obtain sufficient experimental information, we combined previous crystallo­graphic data sets of Spy and Im7 6–45 (data set 1), both the residual electron density and the iodine signals, with the data presented here (data set 2). All selections include the electron density from data set 1, the iodine anomalous signals from data set 1, except for those arising from L19pI-Phe, and a variable combination of iodine anomalous signals arising from L18pI-Phe (data set 2), L19pI-Phe (data set 1 or 2) or K20pI-Phe (data set 2). To test the predictive power of the ensemble and the coherence of the data set, some iodine anomalous signals not used in the fit were back-predicted from the final ensemble. The initial conformational pool, and the binning of the electron density, were identical to our previous study, as were the parameters used in the selection procedure (Salmon *et al.*, 2018 ▸; Horowitz *et al.*, 2016 ▸). This procedure was chosen to ensure that potential deviations between the two studies would arise only from the newly recorded iodine data set.

### 2.3. Isothermal titration calorimetry   

The binding affinity of Spy H96L for Im7 6–26 peptides was determined by isothermal titration calorimetry (ITC) using a MicroCal iTC200 instrument (Malvern). The thermodynamic parameters upon the titration of Spy H96L (1.25 m*M*) with the Im7 6–26 peptides (0.11 µ*M* for the wild type, 0.055 µ*M* for the Im7 6–26 peptides L18pI-Phe, L19pI-Phe and K20pI-Phe) were measured at 10°C in a buffer consisting of 40 m*M* HEPES–NaOH pH 7.5, 100 m*M* NaCl. For each experiment, an injection volume of 2 µl was used at intervals of 180 s. The ITC thermograms were fitted to a one-site model using the *Origin* software provided with the instrument. The Im7 6–26 peptide binds to Spy H96L with a dissociation constant of 7.8 ± 0.6 µ*M*, which is similar to the dissociation constant of a partially folded variant of Im7 and wild-type Spy. The Im7 6–26 peptides L18pI-Phe, L19pI-Phe and K20pI-Phe bind to Spy H96L with dissociation constants in the same range as the wild-type Im7 6–26 peptide (Supplementary Fig. S1).

## 3. Results   

### 3.1. Data-collection strategy   

Spy is one of a group of chaperones that allow clients to fold while remaining continuously bound to the surface of the chaperone (Horowitz, Koldewey *et al.*, 2018 ▸). We have previously observed that the chaperone substrate Im7 exists in a heterogeneous, largely disordered crystallographic ensemble while bound to the chaperone Spy (Horowitz *et al.*, 2016 ▸). To refine strategies for detecting anomalous scattering for observing partially occupied disordered states, we co-crystallized the tight-binding Spy H96L variant with short peptides derived from its client, Im7. These peptides each had a single iodophenylalanine substitution at position 18, 19 or 20.

Our data-collection strategy was geared to determining the positions of these iodines with maximum sensitivity while ensuring that the signals were owing to iodine and not to other weak anomalous scatterers present in the crystal. To maximize the signal intensity, we selected data-collection wavelengths that should give complementary data based on the change in anomalous scattering around the iodine *L* edges (Supplementary Fig. S2). We collected data primarily at λ = 2.3843 Å (*E* = 5.2 keV), just above the *L*_I_ absorption edge of iodine, as well as below the *L*_III_ edge at λ = 2.7552 Å (*E* = 4.5 keV). At these two energies, the anomalous contributions *f*′′ to the scattering factor differ by a factor of 3.9 (*f*′′_4.5 keV_ = 3.42 e^−^ versus *f*′′_5.2 keV_ = 13.41 e^−^). By collecting data in this fashion, we reasoned that we should be able to specifically distinguish anomalous signals of iodine from other elements by analyzing the resulting phased anomalous difference Fourier maps. Peaks that are present in the higher energy data set (above the iodine edge; λ = 2.3843 Å, *E* = 5.2 keV) but absent in the lower energy data set (below the iodine edge; λ = 2.7552 Å, *E* = 4.5 keV) originate from I atoms. We further reasoned that one of the best ways to distinguish noise from real signals is to show that the same anomalous signal exists in independent data sets collected at different crystal orientations. We thus collected data sets at λ = 2.3843 Å (*E* = 5.2 keV) at different goniometer κ angles (κ = −40°, φ = −70° and κ = −20°, φ = −70°) so that we could compare and stringently determine the number and positions of the iodine peaks. Finally, when possible, we compared data from multiple crystals with the same components to reveal the reproducibility of the anomalous signals. To help to identify zinc and chlorine sites in the crystal, we also collected data sets at λ = 4.3200 Å (*E* = 2.87 keV) and λ = 4.5085 Å (*E* = 2.75 keV), just above and below the chlorine *K* absorption edge (*E* = 2.822 keV), respectively. Despite the very long wavelengths, the data quality was good enough to observe the peaks expected for chlorine and zinc ions (Table 3 ▸, Supplementary Fig. S3). To our knowledge, these are the first protein crystallography data sets collected around the chlorine absorption edge.

For the initial inspection of the 5.2 keV phased anomalous difference Fourier maps calculated with initial phases from molecular replacement, a cutoff of 4σ was used, based on the *ANODE* recommendations. Using the refined, final model phases, all putative peaks that were present in the 5.2 keV map above 4σ increased to be at least 6σ, and where possible the threshold was set as high as possible. For example, in the L19pI-Phe data collection, which showed several low-occupancy anomalous signals, the *ANODE* anomalous peaks were analyzed with a cutoff at 6σ to ensure confidence in assigning the anomalous signals beyond the noise level (Supplementary Fig. S5).

It is generally accepted that the rate of radiation damage in protein crystals is proportional to the dose. Here, the effect of radiation damage on crystals has been investigated by monitoring a number of parameters as a function of the absorbed dose (Supplementary Table S1). For each data set, the absorbed dose was estimated using knowledge of the incident-beam characteristics (energy, flux and size) and the crystal characteristics (volume, morphology, unit-cell size, protein atomic content, number of amino acids and solvent composition), as well as the exposure time per image and the total number of images. In the case of multiple and sequential data sets collected from the same crystal, the cumulative dose was evaluated.

### 3.2. L19pI-Phe demonstrates multiple binding poses of Im7 on Spy   

**Figure 1:**
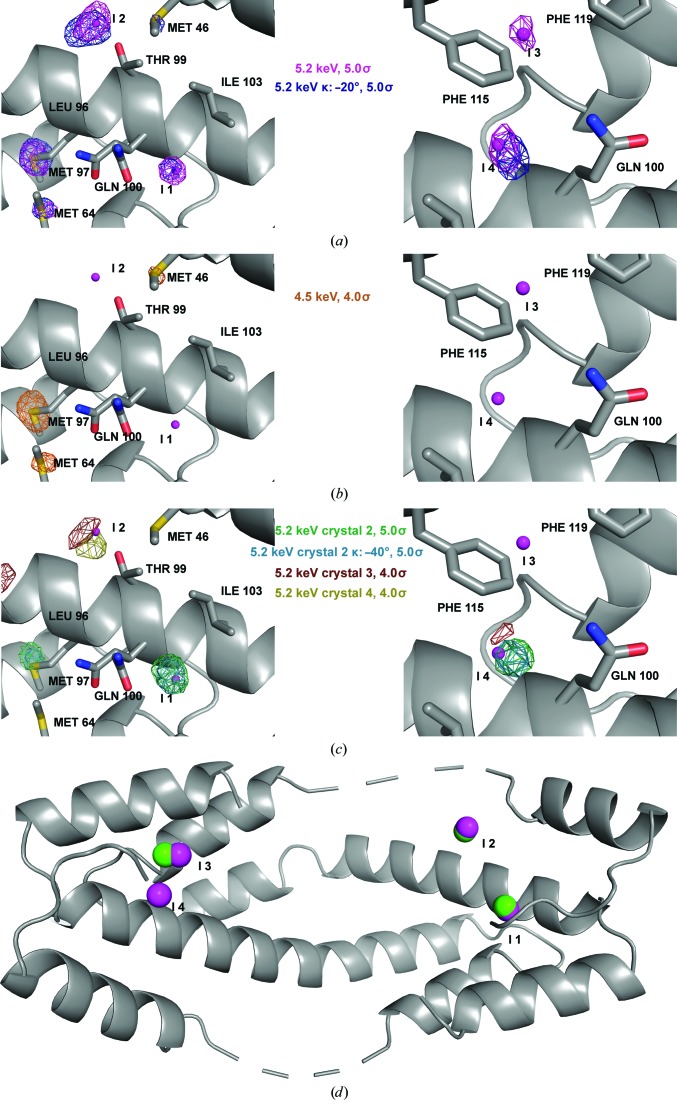
Phased anomalous difference Fourier maps and positions of the four iodine signals in L19pI-Phe crystals, displayed as purple spheres in each panel. Named and numbered residues are from Spy, whereas the partially occupied iodines are labeled I. (*a*) Phased anomalous difference Fourier maps from 5.2 keV data collected at two different goniometer κ angles, contoured at 5σ. (*b*) Phased anomalous difference Fourier map from 4.5 keV data, contoured at 4σ. (*c*) Phased anomalous difference Fourier maps from data collected from different crystals, contoured at 5σ (crystal 2) and 4σ (crystals 3 and 4). (*d*) Overlap of the L19pI-Phe iodine positions (magenta) detected here with the closest iodine signals from previous data (green; Horowitz *et al.*, 2016 ▸).

Of the three peptides tested, the phased anomalous difference Fourier maps of L19pI-Phe show the highest number of anomalous signals that we can attribute to iodine. In addition to the anomalous density attributed to zinc, chlorine and methionine, there are four distinct anomalous signals above 6σ in the phased anomalous difference Fourier maps at 5.2 keV (Fig. 1 ▸ *a* and Table 4 ▸). Of these four peaks, three (iodines 1, 2 and 4) are very well validated by additional maps, as discussed below, whereas the remaining peak is less well supported (Fig. 1 ▸ *c*).

**Figure 2:**
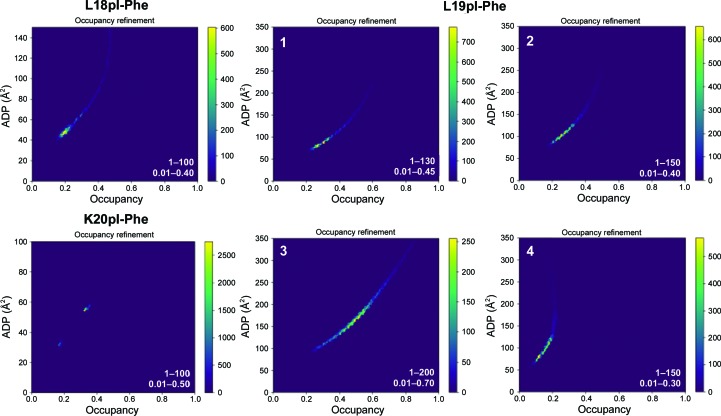
2D histogram showing occupancy refinement of the iodine positions in the three structures, L18pI-Phe (PDB entry 6owx), L19pI-Phe (PDB entry 6owz) and K20pI-Phe (PDB entry 6owy), after 50 rounds of refinement from 10 000 randomly assigned ADP/occupancy starting values (shown in Supplementary Fig. S3). The color scale represents the number of structures in the bin. The starting-value ranges for ADP and occupancy are shown in the bottom right corner of each plot.

When refined from many different starting values for occupancies and atomic displacement factors (Langan *et al.*, 2018 ▸) to estimate the occupancies and temperature factors for these atoms, these iodines displayed both low occupancy and high temperature factors, with occupancies estimated to range from 0.12 to 0.49 and *B* factors ranging from 80 to 164 Å^2^ (Fig. 2 ▸ and Supplementary Fig. S4).

To check whether these signals are attributable to iodine, we collected an additional data set from the same crystal at 4.5 keV, below the iodine *L* edges. At this energy, we would expect that the anomalous signal derived from iodine would dramatically decrease, whereas the change in the anomalous signal from methionine, zinc and chlorine would be negligible. The phased anomalous difference Fourier maps at this energy confirm that the non-iodine scatterers continue to display strong anomalous signals, whereas the signals from the four putative iodines disappear (Fig. 1 ▸ *b*). This experiment indicates that the four anomalous signals are from the Im7 peptides and not from other anomalous scatterers. Moreover, the high σ values of the anomalous signals, the smallest of which was detected at 6.3σ, make it unlikely that these signals represent noise. As a counterexample, the highest peaks not attributable to known anomalous scatterers, which hence could be noise, have σ values of 4.2 in the same map (Supplementary Fig. S5*a*). However, a rigorous way to exclude the possibility that the anomalous signals do represent noise would be to determine whether they are present at the same positions in an additional data set collected at the same energy (at 5.2 keV) but using a different goniometer κ angle (κ = −20°). Anomalous signals that appear in the same positions are highly likely to be true anomalous signals. It is worth mentioning that, after a complete set of data, the intensity of the anomalous signal will decrease owing to radiation damage. Therefore, only the signals with the highest intensity are detected after multiple additional data collections from the same crystal. The phased anomalous difference Fourier maps from this additional data collection showed that three of the anomalous positions from the first data collection are preserved in the data collected at the different κ angle (Fig. 1 ▸ *a*).

Finally, to test whether these anomalous positions are reproducible in other L19pI-Phe crystals, we collected additional 5.2 keV data sets from three other L19pI-Phe crystals. These crystals diffracted to resolutions of 2.2, 2.9 and 3.2 Å, respectively. In the phased anomalous difference Fourier map of the crystal that diffracted to 2.2 Å resolution, we were able to identify three different iodine signals that overlap with the signals detected in the highest resolution crystal (Fig. 1 ▸ *c*). These signals were also present in other data collections (κ = −40°) from the crystal that diffracted to 2.2 Å resolution (Fig. 1 ▸ *c*). In the crystals that diffracted to 2.9 and 3.2 Å resolution, we only detected one iodine signal in each, but these overlapped well with the strongest anomalous signal from the highest resolution crystal. It is therefore clear that the ability to detect low-intensity signals is highly dependent on the crystal quality, as the best diffracting crystals showed the most high-quality anomalous signals.

To further examine the anomalous peaks detected here, we also compared these iodine positions with those identified in our previous Spy–Im7 crystallo­graphic study, in which we used the same L19pI-Phe peptide with Spy H96L. We found that three of the four iodine anomalous positions detected here were also detected and used in our previous analysis (Fig. 1 ▸ *d*).

The lowest L19pI-Phe anomalous peak (6.3σ at 5.2 keV) is not present in the anomalous data set from the crystals diffracting to lower resolution. However, this signal is very close (1.3 Å) to an L19pI-Phe anomalous peak observed in our previous Spy–Im7 crystallographic study (Fig. 1 ▸ *d*). So, although this peak was not well supported by the additional data sets within this study, it was detected in our previous study.

### 3.3. Other Im7 peptides suggest additional Im7 binding sites in the crook of the Spy cradle   

**Figure 3:**
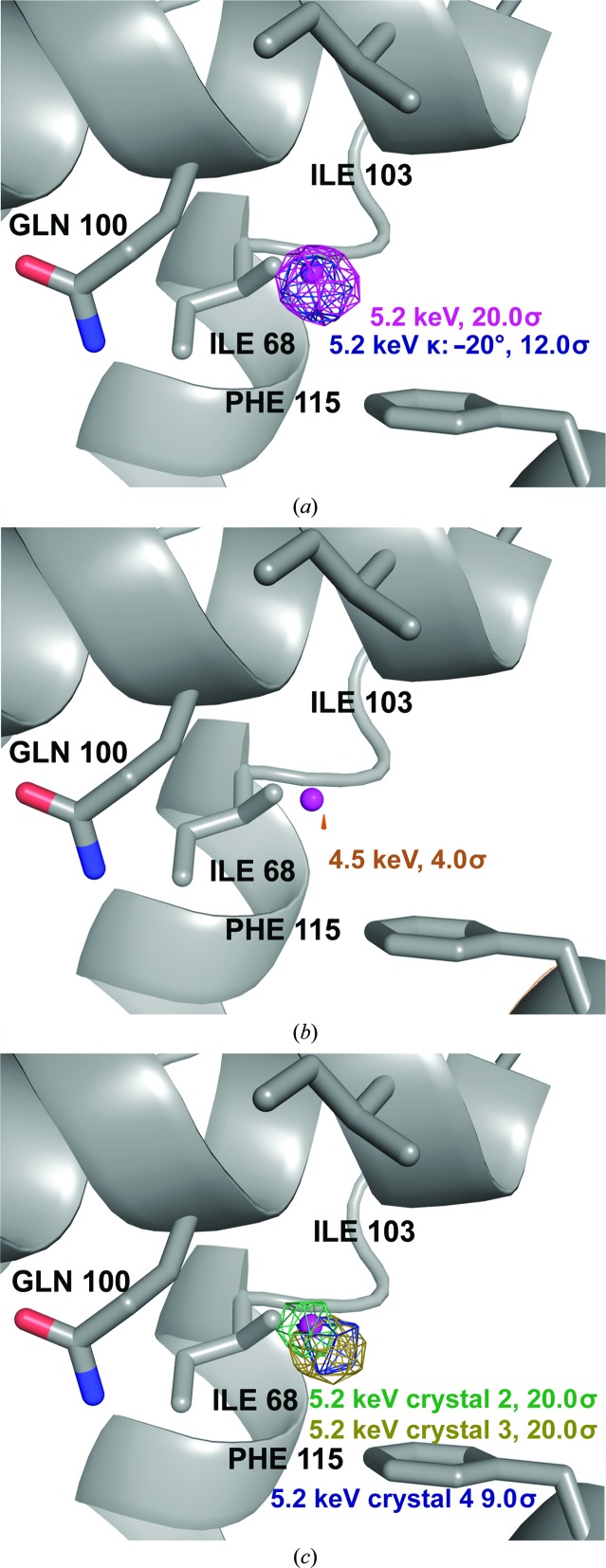
K20pI-Phe phased anomalous difference Fourier maps, with the I atom depicted as a magenta sphere. Named and numbered residues are from Spy. (*a*) Phased anomalous difference Fourier maps from 5.2 keV data sets collected at two goniometer κ angles, contoured at 20σ and 12σ, respectively. (*b*) Phased anomalous difference Fourier map from data collected at 4.5 keV, contoured at 4.0σ. (*c*) Phased anomalous difference Fourier maps from data collected at 5.2 keV from three additional crystals, contoured at 20σ (crystals 2 and 3) and 9σ (crystal 4).

The Im7 peptides L18pI-Phe and K20pI-Phe crystallized in complex with Spy show a very different pattern compared with the multiple signals observed for L19pI-Phe. The 5.2 keV phased anomalous difference Fourier maps of L18pI-Phe and L20pI-Phe each show only one distinct anomalous signal attributable to iodine. In both cases the peak heights in the phased anomalous difference Fourier maps were very strong: the iodine signals were detected at 26.4σ and 32.5σ for L18pI-Phe and K20pI-Phe, respectively (Figs. 3 ▸ and 4 ▸, and Table 4 ▸). Both peaks are in the interior of the cradle of Spy, near the flexible loop region.

For K20pI-Phe, a second data set was collected below the iodine edge at 4.5 keV. The large decrease in the anomalous signal in this data set confirmed that the position ascribed to iodine was indeed iodine (Fig. 3 ▸ *b*). To further validate this observation, we collected a third data set at an additional goniometer κ angle (κ = −20°) above the iodine edge. This data set again showed strong anomalous signal (16.1σ in the phased anomalous difference Fourier map) at the same position (Fig. 3 ▸ *a*), with the peak height reduced by radiation damage (Supplementary Table S1). Finally, we collected data sets at 5.2 keV from three additional crystals that showed strong anomalous signals in the same position (26.7σ, 26.0σ and 6.5σ in phased anomalous difference Fourier maps), demonstrating good reproducibility for this signal (Fig. 3 ▸ *c*). Consistent with the L19pI-Phe data collections, the strength of the iodine anomalous signal detected is highly dependent on the crystal quality. This combination of measurements confirmed that K20pI-Phe produced detectable anomalous scattering from a single position in the crystal, providing an ideal case for determining the position of a partially occupied anomalous scatterer.

**Figure 4:**
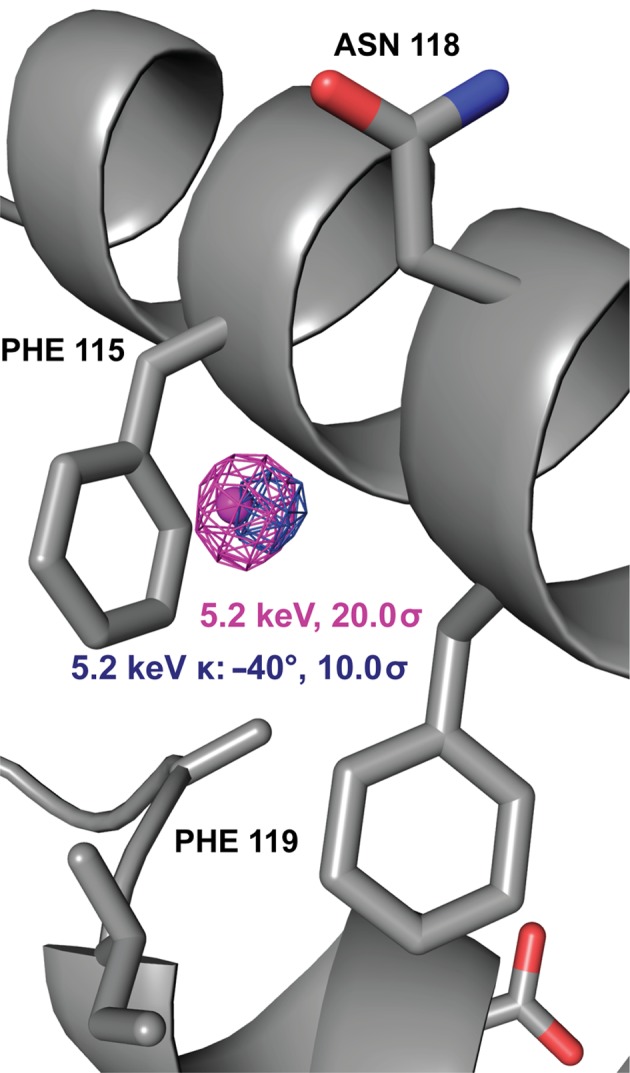
L18pI-Phe phased anomalous difference Fourier maps collected at 5.2 keV at two different goniometer κ angles, with the I atom depicted as a magenta sphere. Named and numbered residues are from Spy.

Unfortunately, for L18pI-Phe the single crystal that showed diffraction adequate for anomalous analysis degraded during the second 5.2 keV data-set collection (Fig. 4 ▸ and Table 5 ▸). This data set, collected at κ = −40°, confirmed the presence of the anomalous signal (Fig. 4 ▸), but the 4.5 keV data set could not be analyzed further owing to radiation damage. However, other indicators strongly suggest that this *ANODE* anomalous peak also arises from iodine. (i) The height of the *ANODE* anomalous peak is the second largest observed in this study and, at 26.4σ in the phased anomalous difference Fourier map, is substantially larger than the signals observed from zinc, chlorine and methionine sulfur, the largest of which is 9.3σ. (ii) The position of this anomalous signal is structurally inconsistent with expected zinc- or chlorine-binding residues, which would be required to enable binding with a suffciently high occupancy to produce a strong anomalous signal. For example, this signal does not occur in the vicinity of zinc- or chloride-binding residues. Thus, although the 4.5 keV data set was not analyzed for this crystal, the strength of the signal and its chemical environment are inconsistent with it being derived from zinc or chloride, making it likely that the anomalous signal is owing to the iodine in the Im7 peptide.

Further support for the L18pI-Phe signal arising from iodine derives from the observation that carbon–iodine bonds are labile and cleavage is expected to occur owing to radiation damage (von Schenck *et al.*, 2003 ▸; Zwart *et al.*, 2004 ▸; Koch *et al.*, 2011 ▸; Ennifar *et al.*, 2002 ▸), a feature that has previously been exploited for phasing purposes (Schiltz & Bricogne, 2007 ▸; Zwart *et al.*, 2004 ▸). Thus, for anomalous signals of peptide-bound iodine, we would expect to see a decrease in signal intensity as a function of increasing X-ray dose, as the iodine is cleaved from the phenyl side chain. This trend is demonstrated not just for L18pI-Phe, but also for K20pI-Phe, in Table 5 ▸.

Examining the radiation dosage, the absorbed doses estimated for the L18pI-Phe, L19pI-Phe and K20pI-Phe crystals are 0.66, 1.35 and 1.43 MGy, respectively (Supplementary Table S1), suggesting that these data are not substantially affected by radiation damage. The greater contribution to the cumulative absorbed dose is mostly made by the dose absorbed during the subsequent data collection at 4.5 keV (8.32 and 8.49 MGy for L19pI-Phe and K20pI-Phe, respectively). Therefore, the anomalous signal peak heights in the later data sets collected at 5.2 keV, in different crystal orientations, are definitively affected by the high cumulative dose during multiple and sequential data collection. The decrease in the anomalous signals observed in the latter data sets is consistent with specific radiation damage suffered by the crystal.

**Figure 5:**
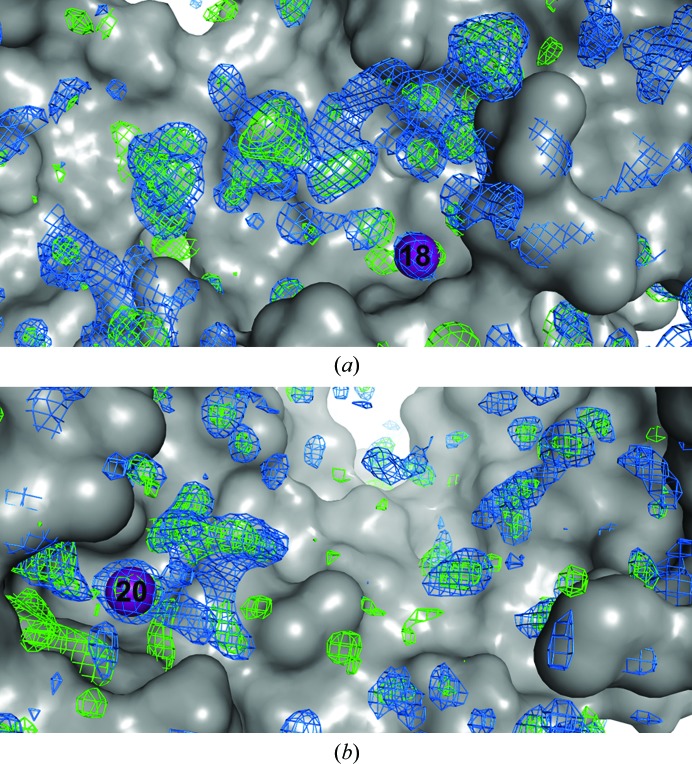
Residual electron density from disordered peptides. (*a*) L18pI-Phe peptide density. (*b*) K20pI-Phe peptide density. The 2*F*_o_ − *F*_c_ map is displayed in blue, contoured at 0.6σ, and the *F*_o_ − *F*_c_ map is displayed in green and red, contoured at 2.5σ. Spy is shown as a gray surface. At this contour level, no negative *F*_o_ − *F*_c_ density is visible in these regions.

The larger peak heights for L18pI-Phe and K20pI-Phe, compared with each of the L19pI-Phe peaks, suggest a higher level of occupancy at these positions. Anomalous site refinements show that the occupancies of L18pI-Phe and K20pI-Phe are approximately 20% and 33%, with *B* factors of approximately 47 and 55 Å^2^, respectively (Table 4 ▸). Similarly, the area adjacent to the anomalous signals in L18pI-Phe and K20pI-Phe shows electron density in 2*F*_o_ − *F*_c_ and *F*_o_ − *F*_c_ maps consistent with the presence of a partially occupied, dis­ordered peptide (Fig. 5 ▸). Attempts to perform traditional model building using the residual L18pI-Phe and K20pI-Phe peptide density were unsuccessful owing to the weak nature of the electron density, as well as its somewhat amorphous shape, which is likely to be owing to multiple conformations within the peptide. This density, however, still shows greater residual peptide occupancy than the areas surrounding the multiple L19pI-Phe iodine anomalous peaks, where we failed to see density above background, consistent with the higher occupancy of the single L18pI-Phe and K20pI-Phe positions. Of note, the electron density for the L18pI-Phe and K20pI-Phe mutants has a substantially more defined shape and signal strength than that observed previously in other mutants (Horowitz *et al.*, 2016 ▸).

### 3.4. READ selection using the novel data sets   

**Figure 6:**
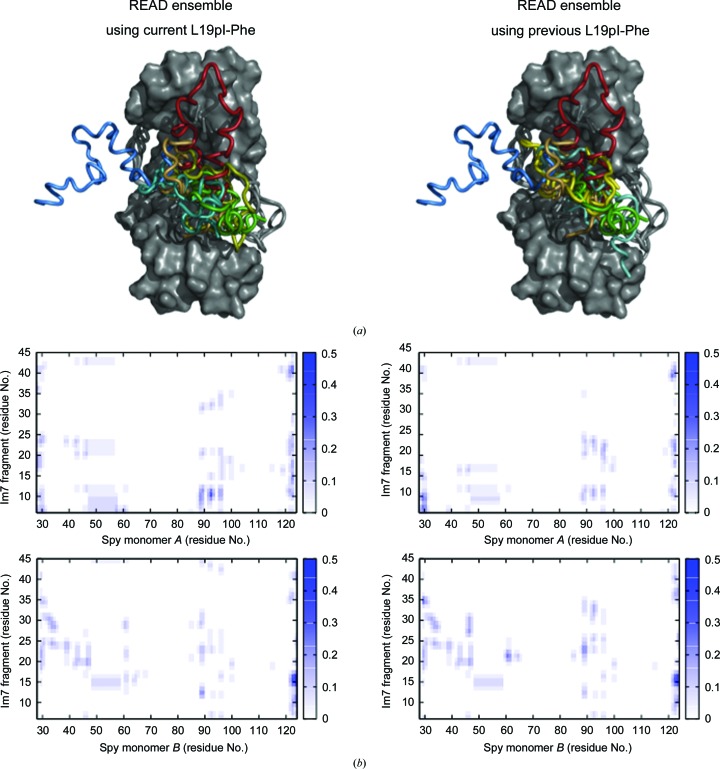
Comparison of the selected ensembles obtained using the current (left) and previous (right) L19pI-Phe data sets. For previously determined iodine positions, please see Supplementary Fig. S10. (*a*) Comparison of the READ ensembles, including the Spy (gray) and Im7 conformations (colors). (*b*) Contact map of the Im7–Spy interactions. The probability of contact increases from white to dark blue.

The previously described READ selection method (Salmon *et al.*, 2018 ▸; Horowitz *et al.*, 2016 ▸) was applied by combining our previous data sets with the new data. For the most direct comparison, we substituted our previously recorded L19pI-Phe signals with the new corresponding data (Section 2 and Supplementary Fig. S9). On average, this procedure was able to fit the L19pI-Phe signals with an average error of 0.37 Å. The results from this selection are highly similar to those previously obtained (Horowitz *et al.*, 2016 ▸), confirming the previously proposed ensemble. The improvement in the detection of L19pI-Phe signals also translates into a higher contribution in the target function of the fitting procedure. Despite little rebalancing of the relative contribution of the data, the analysis leads to essentially the same ensemble (Fig. 6 ▸).

To further investigate the coherence of the two data sets, we tested how an ensemble determined using either the old or the new L19pI-Phe data set could predict the value from the other corresponding data set. Using all of the old data (Horowitz *et al.*, 2016 ▸), the four new L19pI-Phe signals presented here could be predicted with an average deviation of 0.85 Å. Replacing the old L19pI-Phe signals with the new signals, the old L19pI-Phe signals were back-predicted with an associated error of 0.89 Å on average (only one signal was reproduced with an error of 3.9 Å, while the other six could be predicted within 0.41 Å on average). This cross-validation procedure shows that selections run using either the new or the old L19pI-Phe signals can reproduce all of the signals in the other data set, with the exception of one point of the old data set, underlying the internal coherence of the data.

To further investigate the binding of Im7 onto the surface of Spy, we additionally attempted selections using the L18pI-Phe and K20pI-Phe signals. In the case of L18pI-Phe, the molecular-dynamics simulation used to generate the conformational pool was found to not sample positions encompassing the iodine position in the crystal, preventing any fitting and further analysis of this signal (Supplementary Fig. S10). In this case, improvement of the conformational sampling in the molecular-dynamics simulation will be required to fit this iodine position. In the case of K20pI-Phe, a simultaneous fitting of the new L19pI-Phe and K20pI-Phe signals within 0.43 Å on average was possible. However, cross-validation tests in which the K20pI-Phe signal was held out did not back-predict the K20pI-Phe anomalous signal. This issue could have several sources: (i) the K20pI-Phe signal provides unique information that is not present in the remaining data sets, (ii) the K20pI-Phe data are not fully coherent with the remaining data owing to changes induced by the amino-acid substitution or (iii) the sampling in the initial conformational pool is inadequate to allow this validation. In summary, the L19pI-Phe data are consistent with the previously characterized ensemble, while the L18pI-Phe and K20pI-Phe data will require more advanced conformational sampling to properly assess their coherence with the determined ensemble.

## 4. Discussion   

Our new, optimized data-collection strategy provides an improved method to obtain high-quality anomalous signals with less noise contamination; it also verifies the presence of several of the iodine anomalous signals identified in our previous data sets. Combined, the experiments demonstrate that the Im7 peptide binds to Spy in multiple different binding poses, which are detectable using anomalous scattering. Refining the occupancies of the iodines suggests that we are able to detect iodine anomalous signals at as low as ∼12% occupancy, even with a high temperature factor of 80 Å^2^. These low-occupancy states were confirmed via reproducibility through independent data collections at multiple angles and using separate crystals. The iodine positions are plausible, and on average are located around 3.25 Å from the closest atom (Supplementary Table S2). READ selections demonstrated that the new L19pI-Phe data produced a consistent ensemble with previous efforts, while the consistency of the L18pI-Phe and K20pI-Phe data could not be assessed properly with our initial sampling.

Spy–Im7 binding has recently received attention as a model system for understanding chaperone–client interactions. NMR spectroscopy, molecular-dynamics simulations, chemical kinetics and X-ray crystallography have all concluded that client binding to Spy is dynamic and that Im7 can bind to Spy in an array of conformations and poses (He & Hiller, 2018 ▸; He *et al.*, 2016 ▸; Stull *et al.*, 2016 ▸; Horowitz *et al.*, 2016 ▸; Salmon *et al.*, 2016 ▸). These studies showed that Im7 binding occurs at various sites on the concave surface of the Spy cradle. The work here confirms these findings and provides an avenue to identify binding sites directly with higher sensitivity than was previously possible.

**Figure 7:**
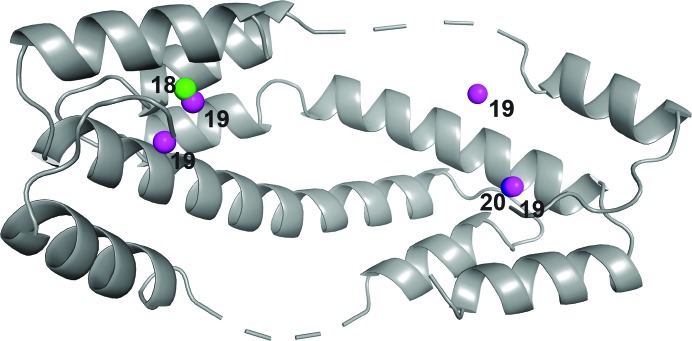
Iodine positions observed in this study, displayed in green for L18pI-Phe, magenta for L19pI-Phe and blue for K20pI-Phe and labeled by Im7 residue number.

The data reported here confirm that Im7 binds to Spy in multiple binding poses, as suggested by the interesting pattern of the anomalous signals (Fig. 7 ▸). The *ANODE* anomalous peaks for L18pI-Phe and K20pI-Phe are both very close to separate L19pI-Phe *ANODE* anomalous peaks. This proximity suggests a degree of promiscuity in the binding sites, in which Im7 can shift small distances to accommodate pI-Phe binding. Moreover, based on the decreasing peak intensities of the iodine anomalous signals during data collection (Table 5 ▸), we can rule out that these positions are iodine ions that have been cleaved from pI-Phe, as this would have produced the opposite trend. These positions are not equivalent, as no iodine density in L18pI-Phe is visible at the K20pI-Phe site or vice versa. These measurements confirm the observation that Im7 binds to Spy promiscuously, but with specific anchor points between Spy and Im7 (Horowitz *et al.*, 2016 ▸; He *et al.*, 2016 ▸).

Direct READ selections and cross-validations demonstrated that the new L19pI-Phe data produced an ensemble consistent with the previously determined ensemble (Horowitz *et al.*, 2016 ▸). Further investigations will be needed to adequately incorporate the analysis of the L18pI-Phe and K20pI-Phe signal positions. At the very least, new methods for increasing the sampling in the initial conformational pool will be required to fit these signals.

In our previous publication (Horowitz *et al.*, 2016 ▸) on crystals of the Spy–Im7 complex, the anomalous data from the partially occupied Im7 conformations were criticized as being too noisy for analysis (Wang, 2018 ▸). In this study, we repeated the measurement of one of the mutants (L19pI-Phe) used in our previous study. Three of the four sites identified here for L19pI-Phe were also observed in our earlier, noisier data (Horowitz *et al.*, 2016 ▸). Although our previous cross-validation analyses demonstrated that even our earlier, noisier anomalous data contained valuable information (Horowitz *et al.*, 2016 ▸; Horowitz, Salmon *et al.*, 2018 ▸) about the Im7 conformations that could then be modeled, the sensitivity of the previously reported experiments was certainly a limiting factor in the technique. In the previous work, the data quality was limited by a combination of detector size and sensitivity owing to the CCD detectors employed, as well as air scattering and absorption. The new long-wavelength beamline I23 at Diamond Light Source enables the imposition of more stringent criteria for the detection of weak anomalous signals. This approach should dramatically improve the ability to delineate disordered molecules in crystals.

## Supplementary Material

PDB reference: Spy H96L, complex with L18pI-Phe Im7 peptide, 6owx

PDB reference: complex with K20pI-Phe Im7 peptide, 6owy

PDB reference: complex with L19pI-Phe Im7 peptide, 6owz

Supplementary Figures and Tables,. DOI: 10.1107/S2059798319014475/wa5123sup1.pdf

## Figures and Tables

**Table:** Values in parentheses are for the outer shell. The space group is *P*4_1_22 in all cases.

	L18pI-Phe	L18pI-Phe, second data set (κ = −40°, φ = −70°)	L19pI-Phe, first crystal	L19pI-Phe, first crystal	L19pI-Phe, first crystal, second data set (κ = −20°, φ = −70°)	L19pI-Phe, first crystal
Wavelength (Å)	2.3843	2.3843	2.3843	2.7552	2.3843	4.5085
*a*, *b*, *c* (Å)	42.80, 42.80, 253.78	42.87, 42.87, 253.97	43.02, 43.02, 257.15	43.01, 43.01, 256.91	43.09, 43.09, 257.62	43.16, 43.16, 257.96
α, β, γ (°)	90.00, 90.00, 90.00	90.00, 90.00, 90.00	90.00, 90.00, 90.00	90.00, 90.00, 90.00	90.00, 90.00, 90.00	90.00, 90.00, 90.00
Resolution range (Å)	42.30–2.11 (2.16–2.11)	42.33–2.23 (2.29–2.23)	43.02–2.00 (2.05–2.00)	43.01–2.01 (2.06–2.01)	42.94–2.11 (2.16–2.11)	43.16–2.95 (3.03–2.95)
Total No. of reflections	233502 (12906)	192411 (9558)	257104 (14125)	221870 (12079)	231087 (12741)	58977 (1694)
No. of unique reflections	15755 (1112)	12296 (826)	17581 (1283)	17223 (1221)	14660 (991)	5755 (389)
*R*_merge_ (%)	0.064 (1.822)	0.115 (1.935)	0.056 (1.794)	0.057 (2.093)	0.065 (1.619)	0.165 (0.816)
*R*_p.i.m._ (%)	0.016 (0.544)	0.028 (0.586)	0.014 (0.543)	0.014 (0.673)	0.016 (0.454)	0.048 (0.403)
〈*I*/σ(*I*)〉	21.0 (1.4)	14.6 (1.2)	26.2 (1.4)	22.8 (1.0)	23.7 (1.5)	10.1 (1.6)
CC_1/2_	1.000 (0.512)	0.997 (0.520)	0.999 (0.552)	0.999 (0.478)	0.998 (0.647)	0.993 (0.604)
Completeness (%)	99.9 (99.9)	98.4 (95.1)	100.0 (100.0)	99.7 (99.1)	97.1 (90.9)	98.8 (93.4)
Overall *B* factor from Wilson plot (Å^2^)	47.12	52.82	46.08	49.06	52.68	89.78
Anomalous completeness (%)	99.8 (98.2)	98.3 (94.0)	99.9 (100.0)	99.6 (99.2)	96.0 (89.2)	97.8 (89.2)
Anomalous multiplicity	8.2 (6.3)	8.7 (6.2)	7.9 (5.7)	6.9 (5.1)	8.7 (7.0)	5.6 (2.4)

**Table:** 

	L19pI-Phe, first crystal	L19pI-Phe, second crystal	L19pI-Phe, second crystal, second data set (κ = −40°, φ = −70°)	L19pI-Phe, third crystal	L19pI-Phe, fourth crystal
Wavelength (Å)	4.3200	2.3843	2.3843	2.3843	2.3843
*a*, *b*, *c* (Å)	43.19, 43.19, 258.46	43.01, 43.01, 259.27	43.12, 43.12, 259.86	42.95, 42.95, 256.87	42.80, 42.80, 253.90
α, β, γ (°)	90.00, 90.00, 90.00	90.00, 90.00, 90.00	90.00, 90.00, 90.00	90.00, 90.00, 90.00	90.00, 90.00, 90.00
Resolution range (Å)	43.08–2.82 (2.90–2.82)	43.01–2.18 (2.24–2.18)	43.31–2.35 (2.42–2.35)	22.05–2.85 (2.92–2.85)	42.32–3.25 (3.34–3.25)
Total No. of reflections	67203 (1798)	215289 (11599)	182649 (10469)	81739 (4032)	52884 (2353)
No. of unique reflections	6518 (413)	13508 (931)	11184 (801)	5570 (295)	3982 (229)
*R*_merge_ (%)	0.158 (1.043)	0.084 (3.695)	0.078 (1.514)	0.093 (1.477)	0.114 (1.181)
*R*_p.i.m._ (%)	0.044 (0.518)	0.021 (1.055)	0.019 (0.424)	0.024 (0.386)	0.030 (0.335)
〈*I*/σ(*I*)〉	10.4 (1.0)	18.9 (0.7)	21.2 (1.6)	19.2 (1.2)	14.6 (1.8)
CC_1/2_	0.992 (0.523)	0.999 (0.623)	0.998 (0.763)	0.999 (0.673)	0.998 (0.583)
Completeness (%)	98.6 (88.7)	97.8 (94.1)	99.8 (100.0)	87.5 (66.6)	92.9 (75.0)
Overall *B* factor from Wilson plot (Å^2^)	90.89	46.47	56.13	79.59	89.38
Anomalous completeness (%)	97.9 (86.7)	97.0 (91.6)	99.8 (100.0)	81.8 (61.1)	85.5 (67.8)
Anomalous multiplicity	5.5 (2.3)	8.8 (6.6)	9.0 (6.9)	14.7 (13.7)	7.9 (6.0)

**Table:** 

	K20pI-Phe, first crystal	K20pI-Phe first crystal	K20pI-Phe, first crystal, second data set (κ = −20°, φ = −70°)	K20pI-Phe, second crystal	K20pI-Phe, third crystal	K20pI-Phe, fourth crystal
Wavelength (Å)	2.3843	2.7552	2.3843	2.3843	2.3843	2.3843
*a*, *b*, *c* (Å)	42.84, 42.84, 257.63	42.86, 42.86, 257.51	42.91, 42.91, 258.00	42.99, 42.99, 259.38	42.69, 42.69, 255.89	42.59, 42.59, 254.90
α, β, γ (°)	90.00, 90.00, 90.00	90.00, 90.00, 90.00	90.00, 90.00, 90.00	90.00, 90.00, 90.00	90.00, 90.00, 90.00	90.00, 90.00, 90.00
Resolution range (Å)	42.84–2.07 (2.12–2.07)	42.86–2.13 (2.19–2.13)	42.91–2.23 (2.29–2.23)	43.23–2.10 (2.15–2.10)	42.65–2.19 (2.25–2.19)	42.48–3.34 (3.43–3.34)
Total No. of reflections	222445 (11148)	176931 (8429)	157318 (8988)	199534 (9497)	211390 (12146)	53111 (2492)
No. of unique reflections	15762 (1115)	14248 (1015)	11268 (880)	14880 (1022)	13281 (977)	3557 (193)
*R*_merge_ (%)	0.080 (1.483)	0.083 (2.003)	0.075 (1.815)	0.096 (1.019)	0.063 (1.544)	0.076 (1.283)
*R*_p.i.m._ (%)	0.020 (0.455)	0.022 (0.669)	0.019 (0.548)	0.024 (0.325)	0.015 (0.441)	0.019 (0.336)
〈*I*/σ(*I*)〉	18.9 (1.4)	16.8 (1.2)	17.0 (1.4)	14.9 (1.2)	24.8 (1.6)	24.8 (1.7)
CC_1/2_	0.997 (0.582)	0.999 (0.453)	1.000 (0.568)	0.998 (0.620)	1.000 (0.672)	0.999 (0.743)
Completeness (%)	99.7 (98.3)	97.8 (98.9)	87.6 (97.0)	96.9 (91.8)	100.0 (100.0)	89.5 (71.9)
Overall *B* factor from Wilson plot (Å^2^)	43.20	47.49	50.23	47.75	54.71	105.76
Anomalous completeness (%)	98.7 (94.9)	96.2 (97.1)	83.2 (93.8)	95.5 (89.9)	99.8 (100.0)	84.8 (68.1)
Anomalous multiplicity	7.7 (5.3)	6.6 (4.3)	7.5 (5.5)	7.3 (5.0)	8.8 (6.6)	8.8 (7.0)

**Table 2:** Refinement

	L18pI-Phe	L19pI-Phe	K20pI-Phe
PDB code	6owx	6owz	6owy
Resolution range (Å)	42.79–2.06 (2.134–2.060)	42.43–2.05 (2.123–2.050)	42.26–2.07 (2.144–2.070)
Completeness (%)	99.89 (99.87)	99.94 (100.00)	99.65 (99.70)
CC_1/2_	1.000 (0.651)	0.999 (0.861)	0.999 (0.691)
CC*	1.000 (0.888)	1.000 (0.962)	1.000 (0.904)
No. of reflections			
Working set	15669 (1516)	16247 (1538)	15674 (1511)
Test set	996 (93)	976 (92)	939 (91)
Final *R*_cryst_	0.2256 (0.3250)	0.2150 (0.2778)	0.2268 (0.3604)
Final *R*_free_	0.2803 (0.3851)	0.2549 (0.2945)	0.2724 (0.4299)
No. of non-H atoms			
Protein	1373	1382	1403
Ion	15	13	13
Ligand	26	19	16
Water	42	62	58
Total	1456	1476	1500
R.m.s. deviations			
Bonds (Å)	0.011	0.015	0.013
Angles (°)	1.21	1.20	1.23
Average *B* factors (Å^2^)			
Overall	66.97	60.61	58.31
Protein	66.41	60.72	58.74
Ion	81.31	64.32	66.28
Ligand	87.39	92.81	55.76
Water	73.42	54.87	48.21
Ramachandran plot			
Most favored (%)	95.91	95.68	94.71
Allowed (%)	3.51	3.70	4.71
Outlier (%)	0.58	0.62	0.58
Rotamer outliers (%)	0.74	0.70	0
*MolProbity* clashscore	8.89	7.80	4.17
*MolProbity* score	1.76	1.73	1.56

**Table 3:** Peak heights detected for zinc and chlorine from the 2.87 and 2.75 keV phased anomalous difference Fourier maps of the best L19pI-Phe Spy–Im7 crystal (labeled according to PDB entry 6owz) At 2.87 keV, *f*′′ for Cl is 3.98 and *f*′′ for Zn is 4.0. At 2.75 keV, *f*′′ for Cl is 0.43 and *f*′′ for Zn is 4.4 (Supplementary Fig. S2).

Atom	2.87 keV peak height (σ)	2.75 keV peak height (σ)
Zn1	3.6	5.6
Zn2	4.6	5.0
Zn3	5.0	6.1
Zn4	4.2	4.7
Zn5	5.3	7.8
Zn6	6.9	9.6
Zn7	14.0	10.8
Cl1	12.0	0.3
Cl2	11.0	1.1

**Table 4:** Iodine anomalous peak heights, occupancies and *B* factors approximated from repeated refinements (Fig. 2 ▸) for the best crystal of each Spy–Im7 complex at 5.2 keV

Spy–Im7	Peak height (σ)	Occupancy	*B* factor (Å^2^)
L18pI-Phe	26.4	∼0.20	∼47
L19pI-Phe 1	8.9	∼0.31	∼87
L19pI-Phe 2	9.8	∼0.49	∼164
L19pI-Phe 3	6.3	∼0.27	∼106
L19pI-Phe 4	6.4	∼0.12	∼80
K20pI-Phe	32.5	∼0.33	∼55

**Table 5:** Radiation damage specifically affects the iodine anomalous signals As data collection proceeds, the anomalous signals (in σ from phased anomalous difference Fourier maps) for both L18pI-Phe and L20pI-Phe drop while that of a nearby methionine sulfur is less affected.

Spy–Im7	L18pI-Phe		K20pI-Phe	
Residue	Met93	Iodine	Met93	Iodine
First 5.2 keV data set	4.5	26.4	4.9	32.5
Second 5.2 keV data set	4.0	10.6	4.2	16.1
