# Supplementary material for: Identifying dynamic, partially occupied residues using anomalous scattering
Source: Acta Crystallogr D Struct Biol. 2019 Nov 19;75(Pt 12):1084–95. doi: 10.1107/S2059798319014475 (PMC6889914; doi:10.1107/S2059798319014475)
Supplement: Supplementary file 1 [file d-75-01084-sup1.pdf]

STRUCTURAL
BIOLOGY

Volume 75 (2019)

Supporting information for article:

Identifying dynamic, partially occupied residues using anomalous scattering

Serena Rocchio, Ramona Duman, Kamel El Omari, Vitaliy Mykhaylyk, Christian Orr, Zhen Yan, Loïc Salmon, Armin Wagner, James C. A. Bardwell and Scott Horowitz

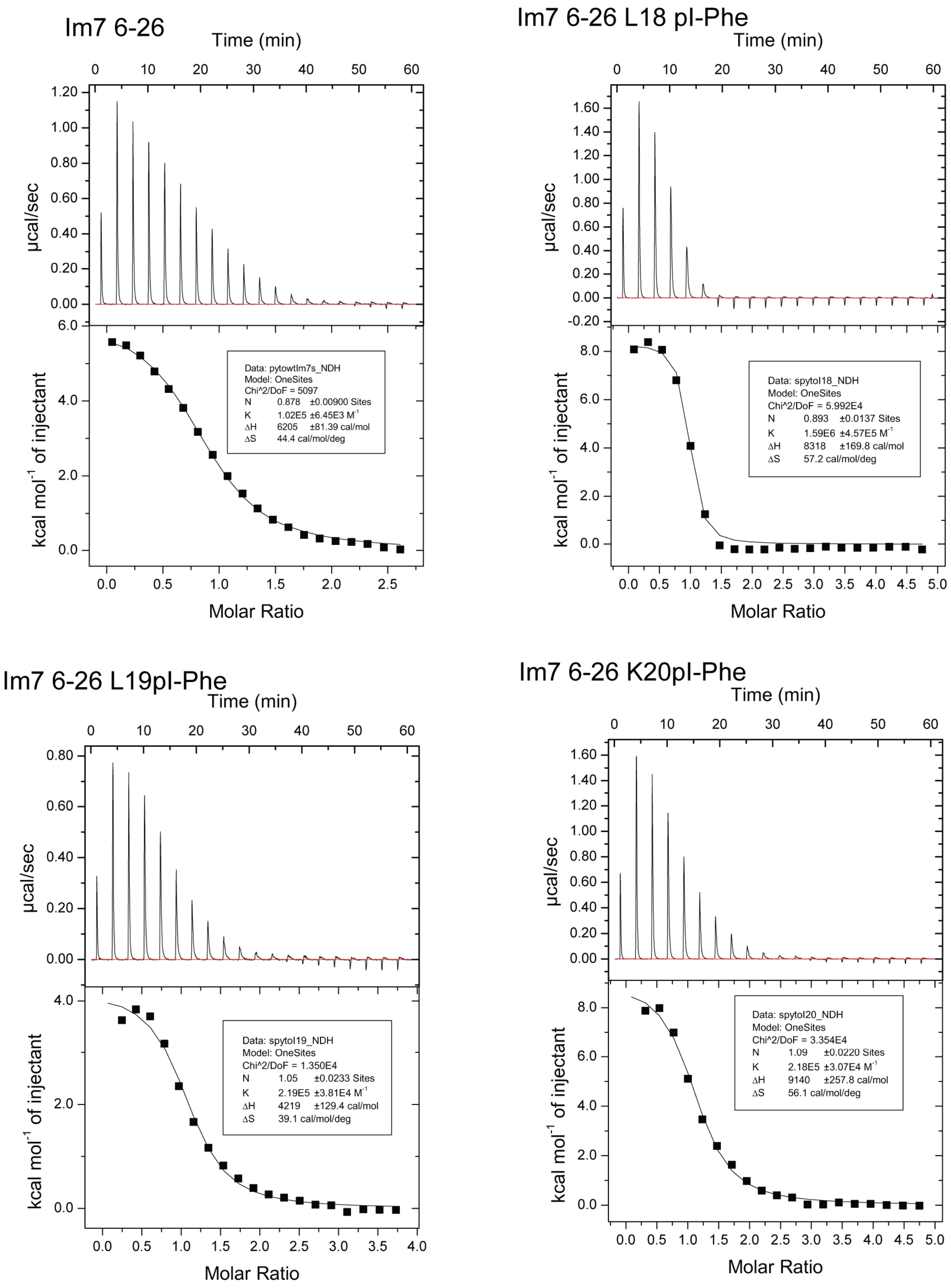

Figure S1 Isothermal titration calorimetry of Spy H96L and Im7 6-26, and its variants studied in this work.

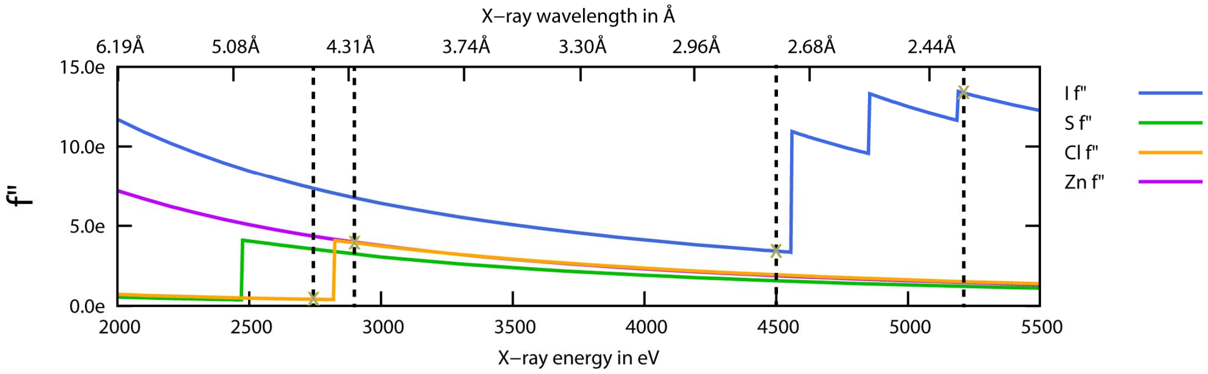

Figure S2 Anomalous scattering plots (f') for atoms of note in the Spy H96L crystals. Data collection energies used are denoted by black dashed bars. Points of interest are denoted with x's.

Table S1 Dose estimation for crystals used in this study, in order of data collection for cumulative dose calculation.

Flux reading error is +/-12%. Italics designate crystals used in refinement, with their respective doses in bold. The increased dose at 4.5 keV is due to two factors. The flux at this energy is 5.2-fold higher than at 5.2 keV. Additionally, X-ray absorption increases significantly towards lower energies. This can also be seen in the 2.75 / 2.87 keV datasets which have ~4.6-fold higher flux values than the 5.2 keV dataset, but because of the larger absorption at lower energy, the dose is around 9-fold higher than at 5.2 keV. The variation in dose for different crystals at 5.2 keV can be accounted to variations of crystal dimensions and shape and the fact that some of the crystals were larger than the X-ray beam.

**Energy (keV)**	**Flux (photons/second) 100 x 100 μm^2 beam**	**Name**	**Crystal Size (μm^3)**	**Dose (MGy)**	**Cumulative Dose MGy**
5200	1.26×10^{10}	*L18pl-Phe*	160 x 70 x 190	0.66	**0.66**
5200	1.26×10^{10}	L18pl-Phe 2nd dataset (k: -40, p: -70)	160 x 70 x 190	0.66	1.33
5200	1.26×10^{10}	*L19pl-Phe*	170 x 20 x 20	1.35	**1.35**
4500	6.65×10^{10}	L19pl-Phe	170 x 20 x 20	8.32	9.67
5200	1.26×10^{10}	L19pl-Phe 2nd dataset (k: -20, p: -70)	170 x 20 x 20	1.35	11.01
2750	5.70×10^{10}	L19pl-Phe	170 x 20 x 20	12.50	23.51
2870	5.90×10^{10}	L19pl-Phe	170 x 20 x 20	12.03	35.55
5200	1.26×10^{10}	L19pl-Phe 2nd crystal	150 x 30 x 20	1.41	1.41
5200	1.26×10^{10}	L19pl-Phe 2nd crystal, 2nd dataset (k: -40, p: -70)	150 x 30 x 20	1.41	2.82
5200	1.26×10^{10}	L19pl-Phe 3rd crystal	100 x 80 x 20	1.64	1.64
5200	1.26×10^{10}	L19pl-Phe 4th crystal	100 x 50 x 20	1.64	3.28
5200	1.26×10^{10}	*K20pl-Phe*	90 x 90 x 50	1.43	**1.43**
4500	6.65×10^{10}	K20pl-Phe	90 x 90 x 50	8.49	9.92
5200	1.26×10^{10}	K20pl-Phe 2nd dataset (k: -20, p: -70)	90 x 90 x 50	1.43	11.35
5200	1.26×10^{10}	K20pl-Phe 2nd crystal	150 x 100 x 20	1.01	1.01
5200	1.26×10^{10}	K20pl-Phe 3rd crystal	140 x 100 x 20	1.44	1.44
5200	1.26×10^{10}	K20pl-Phe 4th crystal	110 x 50 x 20	1.53	1.53

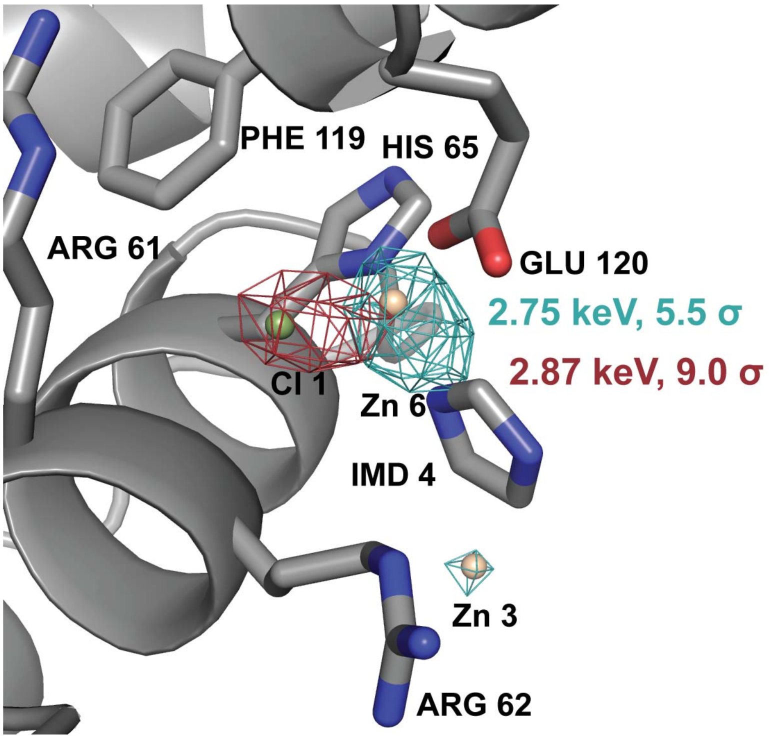

Figure S3 Phased anomalous difference Fourier maps of L19pI-Phe collected at 2.75 and 2.87 keV depicted in cyan and red, and contoured at 5.5 σ and 9 σ, respectively, for identification of chlorine and zinc atoms.

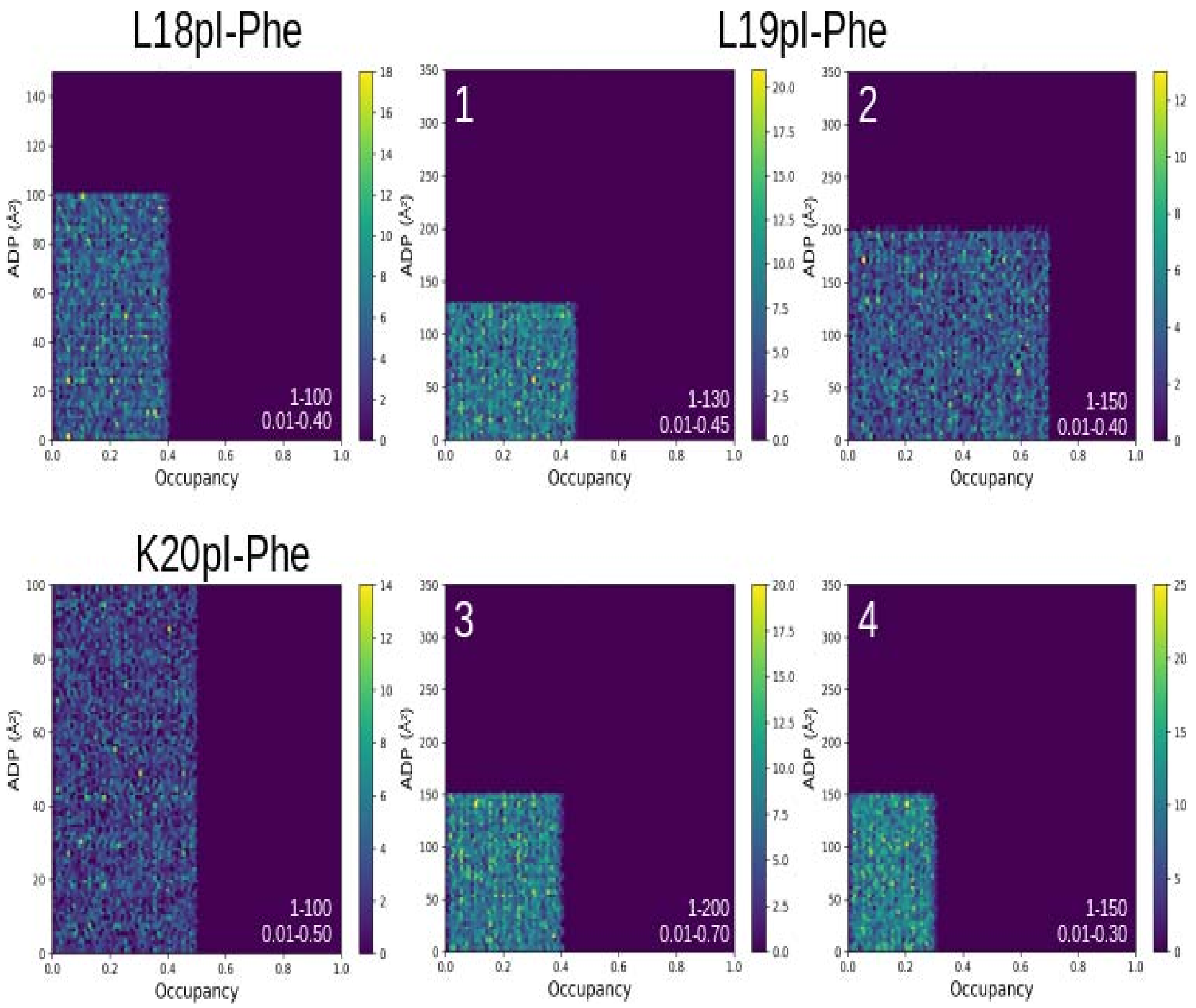

Figure S4 2D histogram showing the 10,000 randomly assigned APD / occupancy starting values for occupancy refinement of the iodine positions in the three structures L18pI-Phe (6OWX), L19pI-Phe (6OWZ) and K20pI-Phe (6OWY). The color scale represents the number of structures in the bin. The starting value ranges for ADP and occupancy are shown in the bottom right corner of each plot.

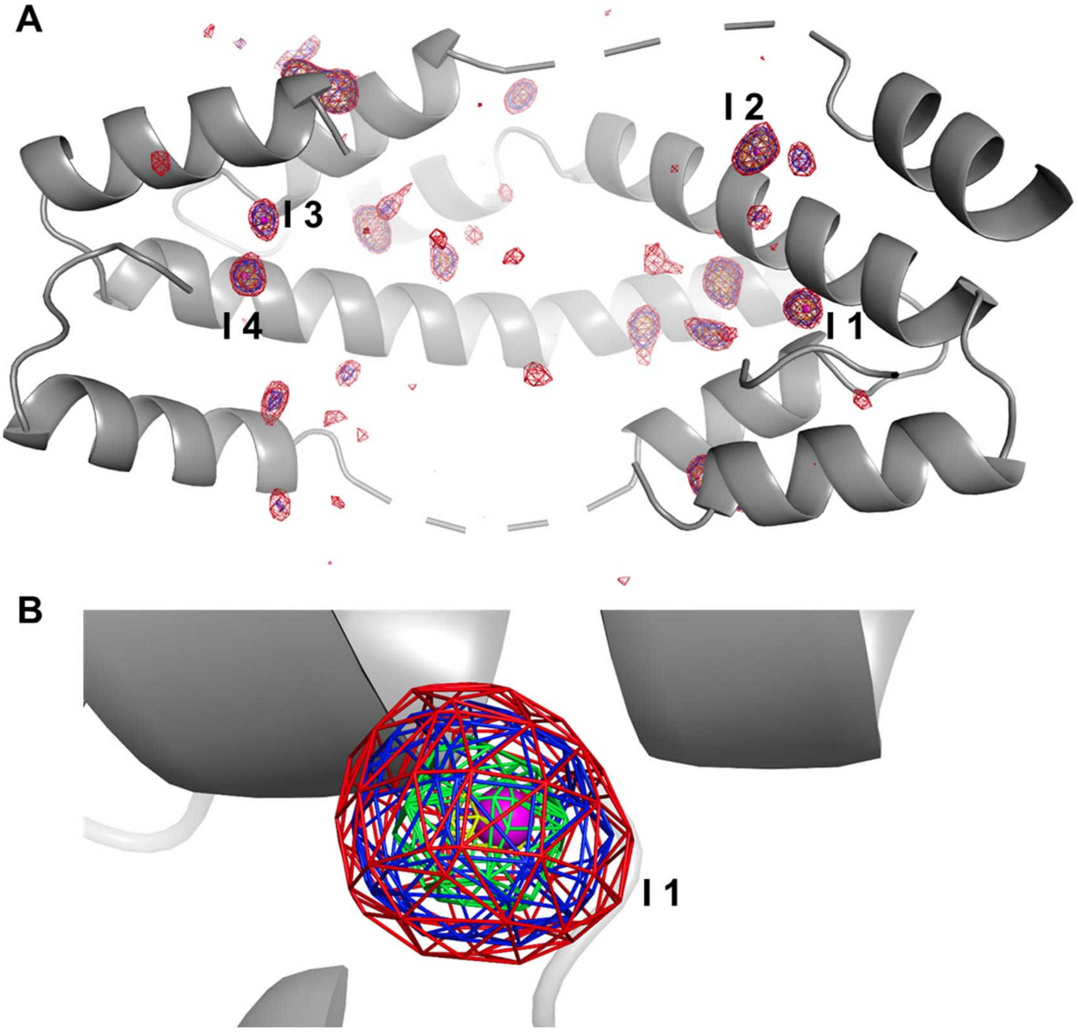

Figure S5 Phased anomalous difference Fourier map of **L19pI-Phe** contoured at various thresholds (red 3.0 σ, blue 4.0 σ, green 6.0 σ, yellow 8.0 σ for (A) the asymmetric unit and (B) one representative iodine. Iodines are displayed as magenta spheres.

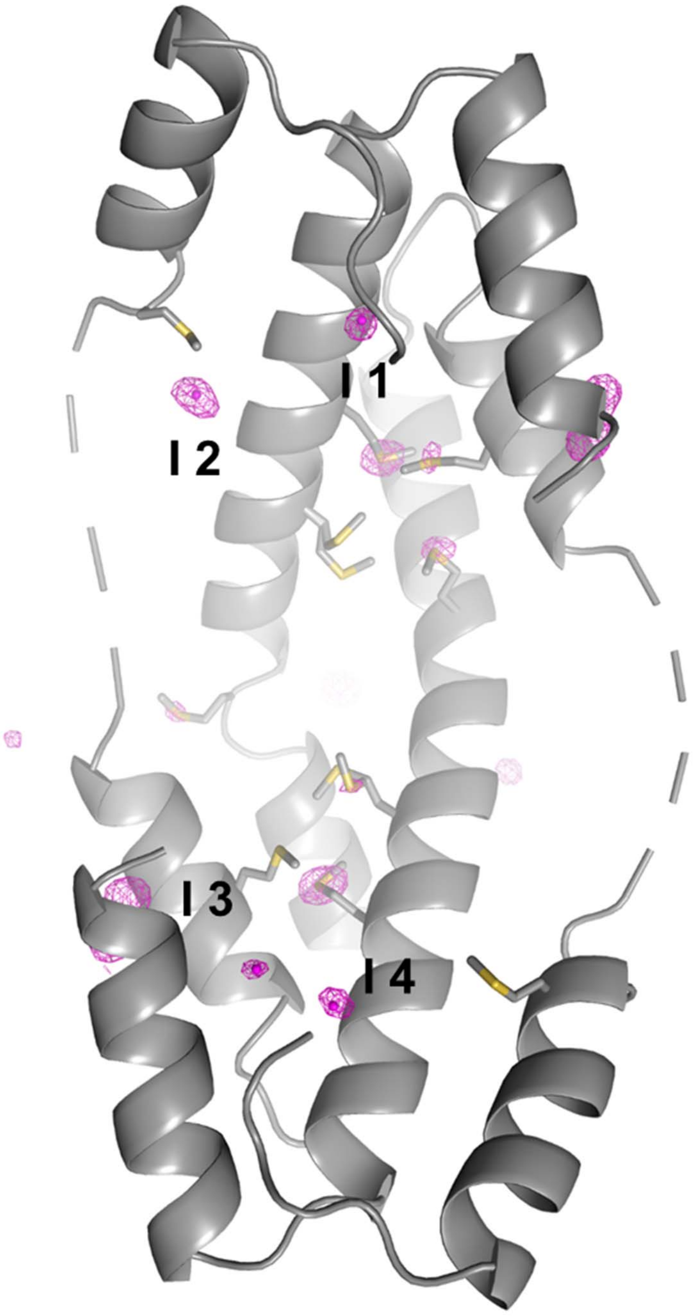

Figure S6 Phased anomalous difference Fourier map of **L19pI-Phe** contoured at 5.0 σ. Iodines are displayed as magenta spheres. Of note, certain anomalous density features visualized here are from neighboring asymmetric units, especially of zinc and chlorine atoms.

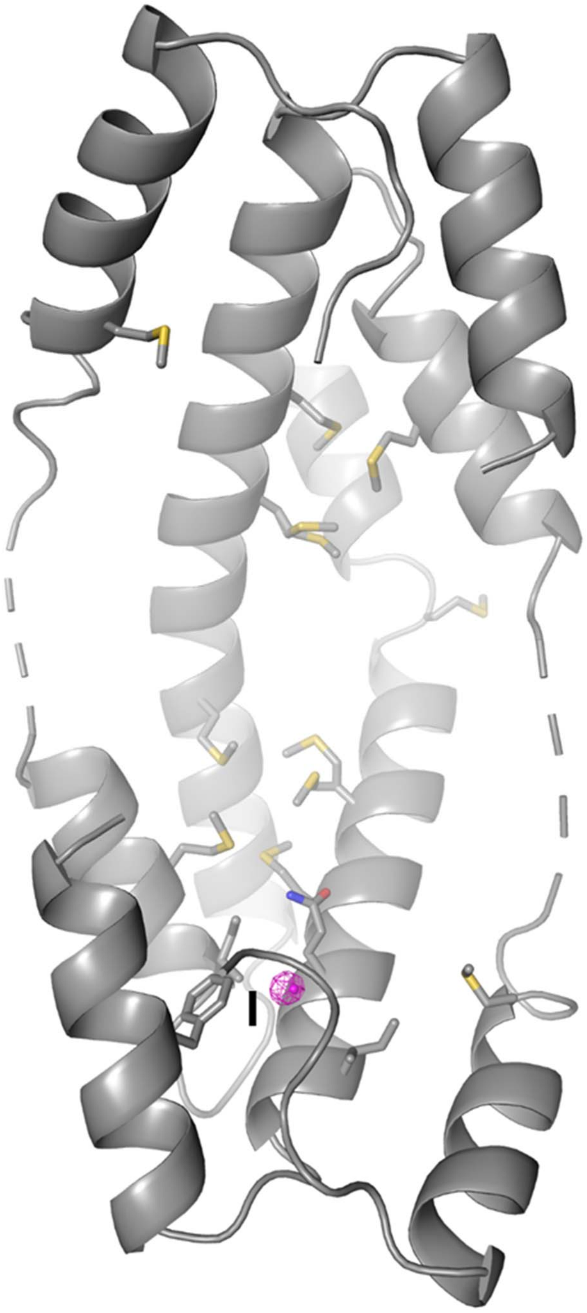

Figure S7 Phased anomalous difference Fourier map of **K20pI-Phe** contoured at 20.0 σ. Iodines are displayed as magenta spheres.

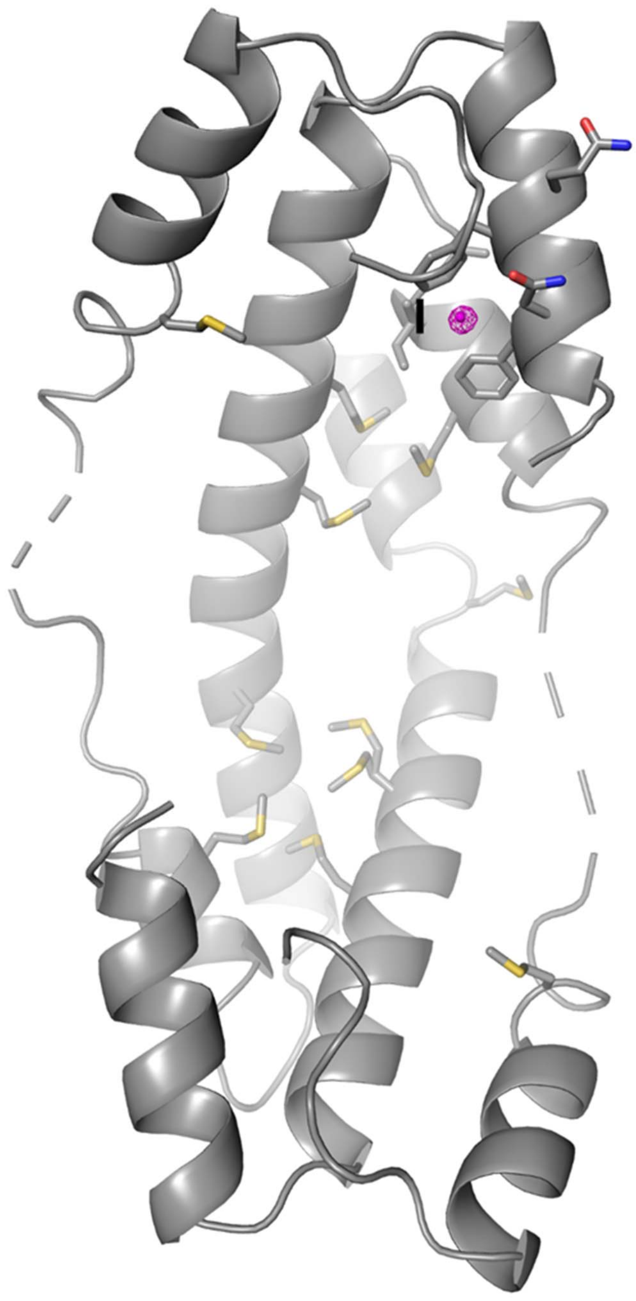

Figure S8 Phased anomalous difference Fourier map of **L18pI-Phe** contoured at 20.0 σ. Iodines are displayed as magenta spheres.

Table S2 Distances from iodines to nearest protein atoms.

Distance (Å) from IOD 205 B (L18pI-Phe)	Atom	Residue
3.39	CE2	115 PHE B
3.65	HE2	
3.42	CD2	
3.67	HD2	
3.08	O	
3.44	HD2	119 PHE B
3.19	HB	
3.36	HA	
3.60	N	
3.58	H	
3.45	HB2	118 ASN B
Distance (Å) from IOD 214 A (K20pI-Phe)	Atom	Residue
3.41	HE1	115 PHE A
3.34	HG2	100 GLN A
3.07	HB3	
3.33	HA	
3.52	O	
3.66	HB	103 ILE A
Distance (Å) from IOD 212 A (L19pI-Phe)	Atom	Residue
3.38	CE2	115 PHE A
2.82	HE2	
3.35	HA,A	110 GLN A
3.37	HA,B	
3.24	HB3,B	
3.39	HB3,A	

3.41	HG2,B	
3.32	CD,A	
2.87	OE1,A	
3.66	HB	103 ILE A
Distance (Å) from IOD 213 A (L19pI-Phe)		
3.58	SD	46 MET A
2.90	HG21	99 THR A
3.63	OG1	
2.84	HG1	
Distance (Å) from IOD 205 B (L19pI-Phe)		
3.28	HD2	119 PHE B
3.41	HE2	115 PHE B
3.45	CE2	
Distance (Å) from IOD 206 B (L19pI-Phe)		
3.21	HG21	103 ILE B
3.61	HB	
3.40	HG2	100 GLN B
2.88	HE21	
3.36	HZ	115 PHE B
3.55	CZ	
2.90	CE2	
1.99	HE2	

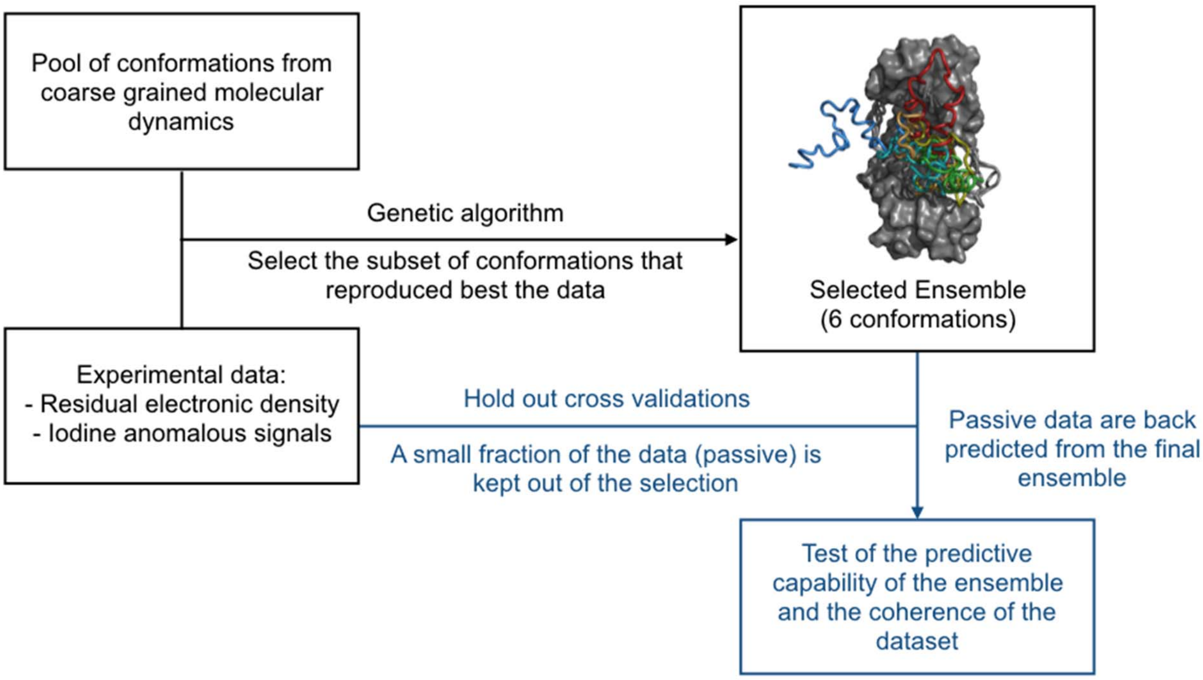

Figure S9 Schematic representation of the READ selection (black) and the cross validation process (blue).

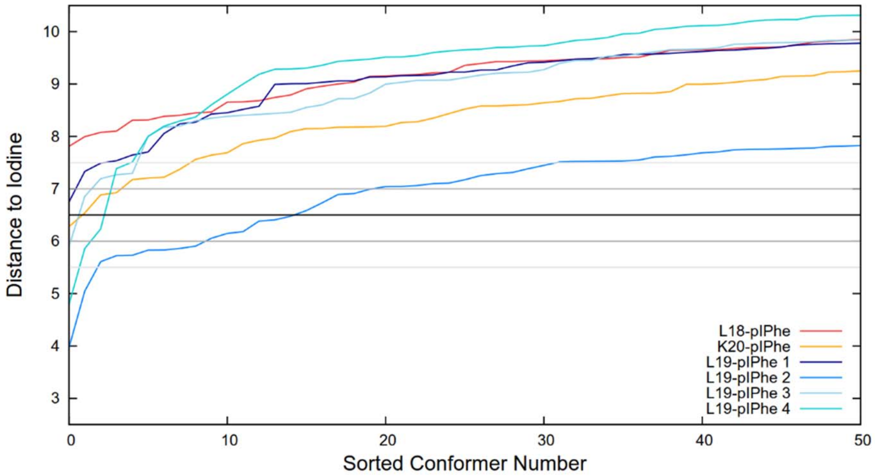

Figure S10 Distance to the iodine anomalous signals during initial pool MD simulation. For each structure in the initial pool, the distance to the iodine anomalous signals were measured. The resulting distances are depicted as colored lines for the 50 conformers with the closest distance to the ideal 6.5 Å for each iodine. Horizontal bars correspond to the expected value of 6.5Å (black), +/-0.5Å (dark grey) and +/-1Å (light grey). The sampling in the MD simulation is insufficient to fit L18pI-Phe, which never comes nearer than 1 Å to ideal positioning (6.5 Å) to the anomalous signal.

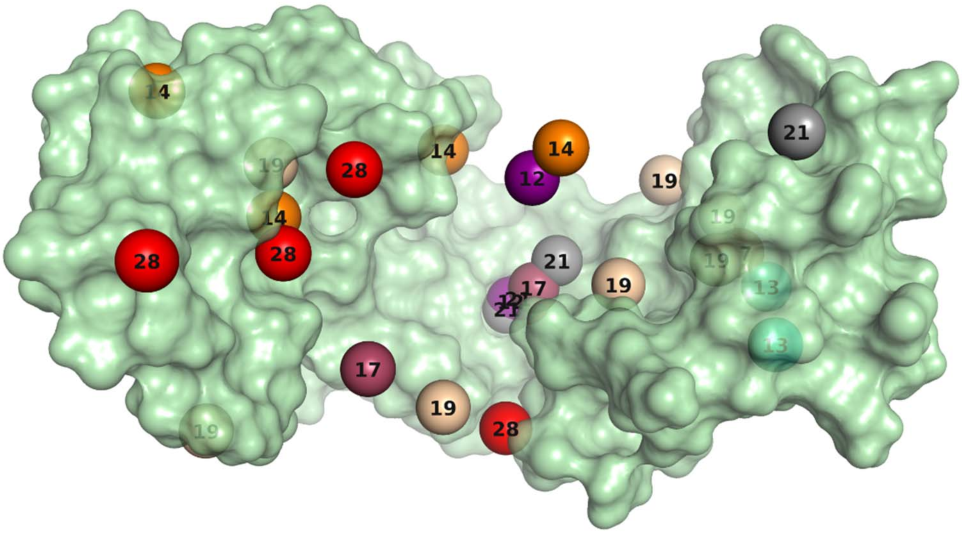

Figure S11 All previously determined iodo-substituted positions for comparison.
